# Is the Reindeer Lichen *Cladonia arbuscula* Really Producing Isousnic Acid? A Chemotaxonomy Query

**DOI:** 10.3390/molecules31010143

**Published:** 2026-01-01

**Authors:** Dagmar Ísleifsdóttir, Maonian Xu, Maia Biwersi, Marie-Jeanne Leblanc, Starri Heiðmarsson, Snæbjörn Pálsson, John L. Sorensen, Elvar Örn Viktorsson, Elín Soffía Ólafsdóttir

**Affiliations:** 1 Faculty of Pharmaceutical Sciences, University of Iceland, Hofsvallagata 53, IS-107 Reykjavik, Iceland; dai8@hi.is (D.Í.); maonian@hi.is (M.X.); mab130@hi.is (M.B.); eov@hi.is (E.Ö.V.); 2 Department of Chemistry, University of Manitoba, Winnipeg, MB R3T 2N2, Canada; leblan38@myumanitoba.ca (M.-J.L.); john.sorensen@umanitoba.ca (J.L.S.); 3 Northwest Iceland Nature Research Centre, Aðalgata 2, IS-550 Sauðárkrókur, Iceland; starri@nnv.is; 4 Faculty of Life and Environmental Sciences, University of Iceland, Sturlugata 7, IS-102 Reykjavik, Iceland; snaebj@hi.is

**Keywords:** chemotaxonomy, *Cladonia arbuscula*, *Cladonia mitis*, DNA barcoding, isousnic acid, lichen, UHPLC

## Abstract

Isousnic acid (isoUA) has been detected in a few usnic acid (UA)-producing lichens with chemotaxonomic values. IsoUA was first isolated from a specimen belonging to *Cladonia arbuscula* s.l. (referred to as *C. mitis* in the publication). However, the isolation and detection of isoUA in this *Cladonia* species have not been reproduced and confirmed with clear evidence. This study focused on *C. arbuscula* s.l. collected in Iceland and aimed to (1) identify the lichen specimen using DNA barcoding and (2) investigate whether isoUA is produced using a series of chromatographic methods. The fungal nuclear ribosomal internal transcribed spacer (nrITS) barcode was sequenced, and the specimen was identified as *C. arbuscula*, following recent circumscription recommendations. Routine metabolite profiling did not detect isoUA, and it could only be identified after vigorous chromatographic purification and concentration steps using flash chromatography and preparative high-performance liquid chromatography. IsoUA was found in trace quantities (~24 µg/g dry weight), which likely explains its absence in routine metabolite profiling. A rapid ultra-high-performance liquid chromatography (UHPLC) method using a pentafluorophenyl column was developed to separate UA and isoUA. Our study highlights the importance of an integrative approach combining DNA barcoding and detailed chromatographic analyses for lichen chemistry research.

## 1. Introduction

Lichens are classic examples of successful microbial symbiosis, adapting to extreme environments and producing a wide range of unique, specialized metabolites [1,2,3]. These lichen-specific compounds, including depsides, depsidones, and dibenzofurans, serve diverse ecological functions, such as protection against UV radiation, herbivory, and allelopathy [4,5,6,7]. They also display significant biological potential, with reported antimicrobial, anti-inflammatory, cytotoxic, and antioxidant activities [3,8,9]. Lichen chemistry played an indispensable role in the early lichen taxonomy before the advent of DNA sequencing, with most morphological species found to be chemically uniform [10]. However, chemical variations can be found within a single lichen taxon, leading to controversy over whether chemical varieties should be treated as separate species or subspecies [11]. Therefore, it is suggested that chemical characters should be combined and correlated with other independent characters for taxonomic investigations [12].

**Figure 1:**
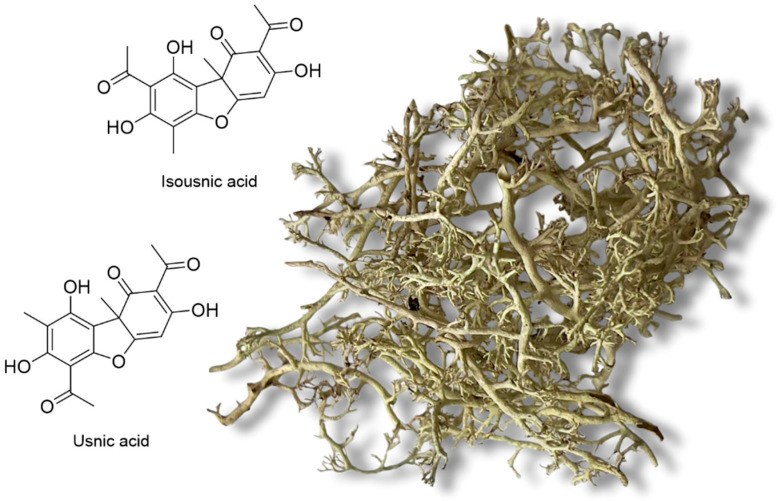
Thallus of the morphologically identified *Cladonia arbuscula* s.l. used in this study and the chemical structures of usnic acid and isousnic acid.

Among the lichen metabolites, the dibenzofuran usnic acid (UA) (Figure 1) is well known for its antibacterial, phytotoxic and insecticidal activity with important ecological roles [13,14,15]. Although UA has occasionally been shown to be accompanied by its structural isomer, isousnic acid (isoUA) (Figure 1), this isomer has not been widely studied for its bioactivity or ecological functions, probably due to its natural paucity. IsoUA has an important chemotaxonomic value in different lichenized fungal lineages, such as the genus *Sphaerophorus* (Sphaerophoraceae) [16] and *Lecanora* (Lecanoraceae) [17].

IsoUA was first isolated from a lichen reported as *Cladonia mitis* by Japanese phytochemists over half a century ago [18]. However, no evidence (e.g., morphological characters according to monographs) was provided for the identification of the sourced lichen materials in that study, and extensive research efforts have been made in relation to the re-circumscription of *C. mitis* [19], which is suggested to be treated as a subspecies of *Cladonia arbuscula* s.l. by Piercey-Normore et al. [20]. In addition, the yield and purification process of isoUA were not described in sufficient detail in the literature to enable reproduction of the results. Since then, the detection of isoUA from this lichen taxon has not been reproduced using chromatographic methods with various sensitivities for purification and detection [18,21,22], except for one putative identification using normal-phase chiral high-performance liquid chromatography (HPLC), which did not include an isolated analytical standard for comparison [23]. Thus, it remains ambiguous whether this lichen truly produces isoUA or not.

The current study focused on the lichen *C. arbuscula* s.l. collected in Iceland and aimed to (1) identify the specimen using DNA barcoding and (2) investigate whether isoUA is present in this lichen using a series of chromatographic and analytical tools. We intended to explore the effectiveness of integrating DNA-based specimen identification with metabolite profiling to unambiguously link the identified metabolites to the phylogenetically identified lichen species.

## 2. Results

### 2.1. Identification of the Lichen Using DNA Barcoding

**Figure 2:**
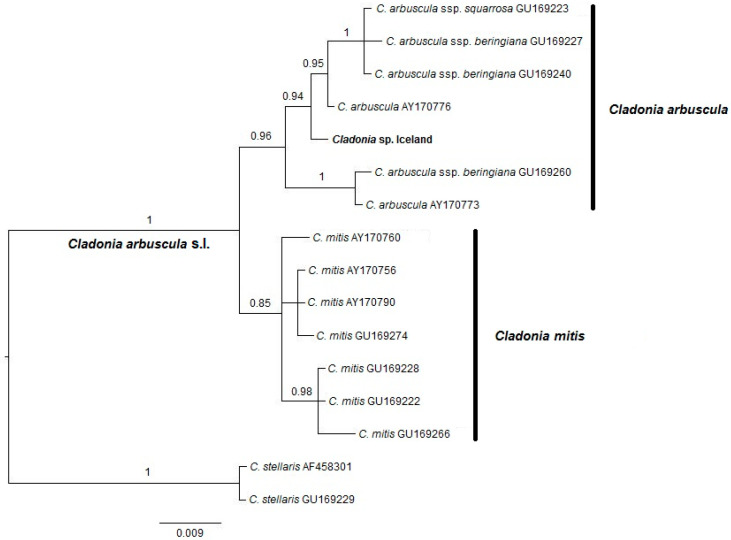
Bayesian tree inferred from the fungal nrITS sequences using the TRNEF+G model. Posterior probability values are marked on major branches. The query sequence obtained from the Icelandic specimen is marked in bold.

To verify the species identity of our Icelandic specimen, we performed DNA-based identification. This is particularly important within the *Cladonia arbuscula* complex, where *C. mitis* and *C. arbuscula* are morphologically very similar and can produce overlapping chemotypes [19,20]. The nrITS sequence obtained from the Icelandic specimen has been deposited in GenBank under accession number PX470057. BLAST search (https://blast.ncbi.nlm.nih.gov/Blast.cgi, accessed on 26 December 2025) revealed two 100% matches to *Cladonia arbuscula* voucher sequences (KY266933 and AY17775), and 99.8% identity with *C. arbuscula* ssp. *beringiana* (GU169252). The sequence also showed 99.1% identity with a *Cladonia submitis* voucher (OL694697) and 98.3% identity with a *Cladonia mitis* strain (OL605254). The BLAST results for the RNA polymerase II second largest subunit (RPB2) sequence (GenBank accession number PX549135) showed the highest identity with *C. submitis* (99.6%; OL963671 and OL963660), followed closely by *C. arbuscula* isolates (99.5%). No *C. mitis* sequences were recovered among the top 50 BLAST hits for RPB2. The Bayesian tree constructed from the fungal nrITS alignment shows two monophyletic clades, consisting of the *C. arbuscula* clade and the *C. mitis* clade. The former shows much higher posterior probability than the latter. It confirms that the Icelandic specimen belongs to the *C. arbuscula* clade (Figure 2). A neighbor-joining tree was also constructed using the nrITS data matrix, which shows the same topology, with our specimen placed in the *C. arbuscula* clade (Figure S1).

### 2.2. Purification and Identification of isoUA from Cladonia arbuscula

For the purification of isoUA, we started with automated flash chromatography, which showed a major peak of UA with some tailing. Fractions that potentially contained isoUA were collected after the UA peak (Supplementary File, Figure S2). To remove the contaminating UA, we continued the purification with semi-preparative HPLC, which provided the baseline separation of UA (19.17 min) and the putative isoUA peak (21.41 min) (Supplementary File, Figure S3). After evaporation, 0.4 mg of isoUA was obtained.

**Figure 3:**
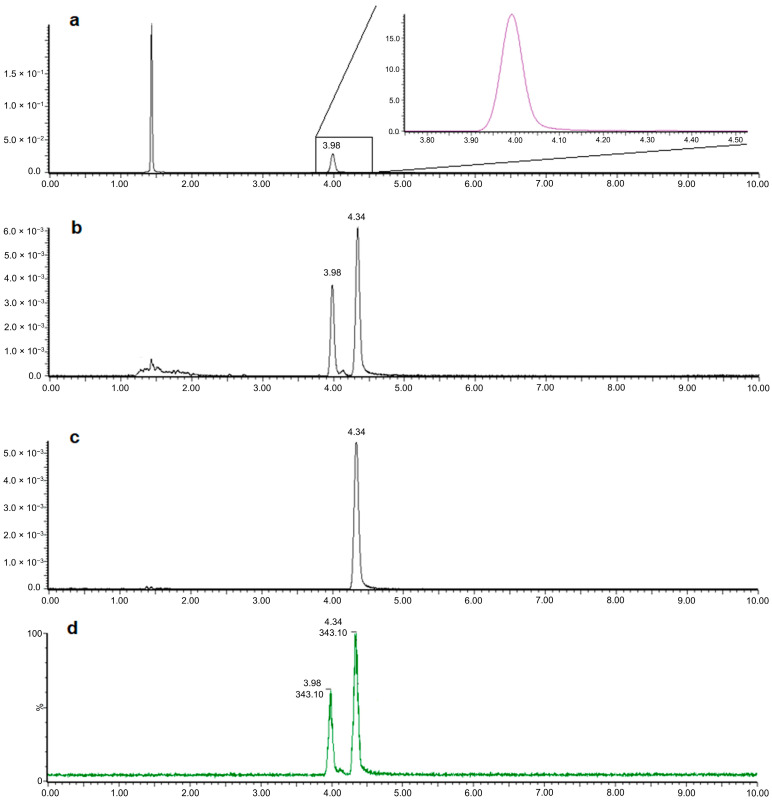
UHPLC separation of usnic acid (UA; t_R_ = 3.98 min) and isousnic acid (isoUA; t_R_ = 4.34 min) of the Icelandic specimen of *C. arbuscula*. Black lines represent UV chromatograms, the purple line shows a magnified UA peak (**a**), and the green line shows extracted ion chromatograms (**d**). UHPLC chromatograms are shown for (**a**) the lichen *C. arbuscula* extract from Iceland showing the UA peak, (**b**) collected effluents from PuriFlash showing the UA and isoUA peaks, (**c**) purified isoUA peak from the semi-preparative HPLC, and (**d**) single ion monitoring of ions at 343.1 *m*/*z* confirming the deprotonated molecular ion of UA and isoUA.

**Figure 4:**
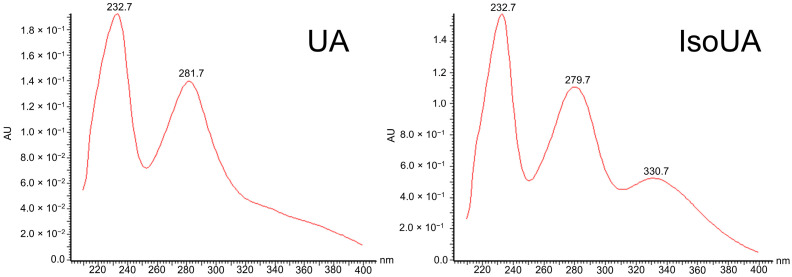
UV spectra of usnic acid (UA) (**left**) and isousnic acid (isoUA) (**right**) from a *Cladonia arbuscula* specimen from Iceland.

Routine ultra-high-performance liquid chromatography–photodiode array detection–mass spectrometry (UHPLC-PDA-MS) profiling of *C. arbuscula* extract showed the presence of UA (t_R_ = 3.98 min), with no sign of isoUA (Figure 3a). However, after combining 34-times collection of effluents (following the UA peak) from the flash chromatography, the isoUA peak became visible at 4.34 min (Figure 3b). With further semi-preparative HPLC purification, isoUA was obtained (Figure 3c) with over 99% purity at 280 nm. Its identity was confirmed by comparing the retention time with a reference lichen (*B. ramuliferum*) that produces isoUA (Supplementary Files, Figure S4), and by MS data (Figure 3d). The high-resolution MS spectrum of the purified isoUA is shown in the Supplementary File, Figure S5. In addition, we importantly observed a third UV absorbance maximum for isoUA at 330 nm, which is not observed for UA (Figure 4).

## 3. Discussion

Accurate identification of lichen or plant material is a prerequisite for the reproducibility of scientific results. It is especially important when it comes to taxa with subtle morphological differences among closely related taxa. The reindeer lichen *C. arbuscula* is taxonomically challenging, as the nucleotide variation in the fungal nrITS locus does not always correlate with morphological variation [19]. Some phylogenetic analyses have indicated a distinct *C. mitis* clade with clear splitting from a more diffuse *C. arbuscula* s.l. clade [20]. We used the same sequence matrix as used in that study to identify our Icelandic *Cladonia* specimen. It was shown to belong to *C. arbuscula* s.l., clearly separated from the circumscribed *C. arbuscula* ssp. *mitis* clade (Figure 2) [20]. We also produced the RPB2 sequence from the specimen with high similarity (99.6% match) to one reference sequence of *C. submitis*. However, the limited availability of *C. arbuscula* RPB2 sequences currently prevents a more comprehensive phylogenetic analysis using this locus. In two phylogenetic studies, *C. submitis* seemed to be more closely related to the *C. arbuscula* s.l. lineage than the *C. mitis* clade [24,25]. DNA barcoding and phylogenetic analysis are feasible and rapid methods for specimen identification, which allows comparison with other reference sequences deposited online. It is especially useful when distinguishing specimens with subtle morphological differences.

Although effective extraction of plant polyphenolics commonly uses aqueous acetone [26], the extraction of UA (and isoUA) requires more lipophilic solvents due to the intramolecular hydrogen bonding that decreases the polarity of the molecule. In this study, ethyl acetate was selected over acetone, as UA exhibits higher solubility in this solvent compared with others [27]. In addition, the use of fully homogenized powdered lichen materials is essential, as it maximizes the extraction yield of UA-like cortical compounds [28].

IsoUA is more lipophilic than UA, as indicated by its faster elution in both silica-based thin-layer chromatography [18] and normal-phase HPLC [14]. Similarly, in our analyses using reversed-phase methods, IsoUA consistently eluted later than UA. Therefore, the identification of isoUA is primarily based on the chromatographic behavior and retention time. In our study, however, we also observed distinct differences in the UV spectra of the two isomers. Notably, isoUA exhibits a unique third UV absorption maximum at 330 nm, which is shown here more clearly than in the UV spectra previously reported by Kinoshita et al. [23]. This characteristic UV signal could serve as a key spectroscopic marker facilitating the rapid chromatographic identification of isoUA in future studies, particularly since UV detectors are standard equipment in most analytical laboratories.

The isoUA in our Icelandic *Cladonia* specimen is produced in a very low quantity of 24 µg/g or 24 ppm, which likely explains the absence of the compound in the recent literature [18,21,22] working on the same or similar lichens. The reference study by Kinoshita et al. [23], which reported the presence of isoUA in *C. mitis*, did not show the chromatogram or specify the amount of isoUA obtained, and therefore, it is hard to compare our results with this reference study. Chromatographic separation with reference to the lichen *B. ramuliferum* and high-resolution mass spectrometry both confirmed the identity of isoUA in our study. However, the low yield and time-consuming repetitive purification process did not allow for alternative structural elucidation using multiple other spectroscopic methods, including but not limited to nuclear magnetic resonance and electronic circular dichroism. The compound from the Icelandic specimen is probably (+)-isoUA, as indicated by the retention time in the chiral HPLC study [23]. From the same study, it appears that the enantiomeric form of isoUA corresponds to the enantiomer of UA produced by the lichen.

IsoUA is known to have important chemotaxonomic values in the lichen genera *Bunodophoron* (Sphaerophoraceae) [16] and *Lecanora* (Lecanoraceae) [17]. Within *Lecanora*, isoUA production is confined to one of the three recognized *Lecanora* species groups, which include *L. albellula*, *L. coniferarum*, *L. laxa*, and several other *Lecanora* species [17]. Its presence can be correlated with the shape of the conidia for species group distinction [17]. In an earlier study, at least three *Bunodophoron* species were found to produce isoUA as a major metabolite, whereas other species lacked it [16]. Although isoUA is also produced in several *Cladonia* taxa [29], its presence and enantiomeric form in chemotaxonomy have not been explored. This calls for a highly efficient and sensitive chiral HPLC method to separate all four enantiomers. Our previously published chiral reversed-phase HPLC method [30] does not allow simultaneous separation of UAs and isoUAs. Therefore, an advanced chiral HPLC method that separates all four isomers (i.e., both enantiomers of UA and isoUA) in a single run is under development in our laboratory.

## 4. Materials and Methods

### 4.1. Lichen Sampling

The lichen (morphologically identified as *Cladonia arbuscula* s.l.) was collected in the northern part of Iceland in Skagafjörður (Arnarstapi, N: 65.5301, W: −19.5154) on 10 October 2024 (Figure 1). It grows on old glacier moraine with little vascular plant cover, along with *Alectoria sarmentosa* ssp. *vexillifera* and *Cetraria* lichens (e.g., *C. islandica*). The voucher specimen is deposited in the herbarium of the Icelandic Institute of Natural History, Akureyri division, with the voucher number LA32065. The lichen specimen, *Bunodophoron ramuliferum* (L45297 TROM), was borrowed from the Tromsø herbarium as a reference lichen, as it produces isoUA as a major metabolite [31].

### 4.2. Identification of the Lichen by DNA Barcoding

Genomic DNA was extracted from 15 mg of thallus tissue following the CTAB protocol [32]. Two barcoding loci, nrITS and RPB2, were amplified using mycobiont-specific primers developed for *Cladonia* lichens [33]. Touchdown PCR amplifications were carried out [34] and sequenced via Sanger sequencing (Macrogen Europe BV, Amsterdam, the Netherlands). The raw reads were trimmed, aligned, and curated in Geneious Prime^®^ 2024.0.5, and the resulting sequences were compared against GenBank references using BLAST. We used the nrITS alignment data matrix from Piercey-Normore et al. [20], which includes worldwide *Cladonia* voucher specimens and separates *C. arbuscula* s.l. and *C. mitis* into monophyletic groups. We then manually aligned our Icelandic sequence to this matrix to determine whether the specimen belongs to *C. arbuscula s.str.* or *C. mitis*. A Bayesian tree was constructed following the procedure we have previously described [34] using the software MrBayes 3.2 [35]. The software MEGA-X version 10.2.2 [36] was used to construct the neighbor-joining tree using maximum composite likelihood method with 500 bootstrap replicates. Following a guideline [37], we used the uncorrected *p*-distance for distance matrix calculations, without any pre-assumed evolutionary model. The neighbor-joining tree was constructed to graphically summarize the genetic distance data. To reduce the risk of specimen misidentification, a Bayesian tree was also constructed following the procedure we have previously described [34]. The evolutionary model was estimated using PartitionFinder2 [38].

### 4.3. Sample Preparation and Purification

A total of 16.5 g of dried lichen material was ground into a fine powder and macerated overnight in ethyl acetate (600 mL). The resulting extract was filtered, evaporated to dryness, and redissolved in a minimal volume of acetonitrile (ACN). To concentrate any isoUA in the extract, we first used automated flash chromatography (puriFlash^®^ 5.250) via with a C18-XS column (15 µm, 250 × 21.2 mm; Interchim, Montluçon, France) to collect any metabolites eluting after the major component UA. The mobile phase consisted of solvent A (ACN + 0.1% formic acid (FA)) and solvent B (H_2_O + 0.1% FA), used in an 80:20 (A:B, *v*/*v*) ratio. The flow rate was set to 16 mL/min, with UV detection at 280 nm. The injection volume was 2 mL. Since isoUA is more lipophilic than UA, all the fractions eluting after the predominant UA peak were collected and pooled for each run. A total of 34 injections were made to concentrate the isoUA using the flash chromatography system (Supplementary File, Figure S2). The solvent was then evaporated, and the residue was redissolved in ACN. As the collected isoUA was contaminated with UA after flash chromatography, we continued with semi-preparative HPLC (Dionex Ultimate 3000; Thermo Scientific, Watham, MA, USA) using a Luna Phenyl-Hexyl column (250 × 10 mm, 5 µm; Phenomenex, Torrance, CA, USA) for further purification of the isoUA. The mobile phase consisted of solvent A (10% ACN/H_2_O + 0.1% FA) and solvent B (ACN + 0.1% FA) in a 30:70 (A:B, *v*/*v*) ratio. The flow rate was 2.5 mL/min, and the UV detection wavelength was set to 280 nm. Samples were dissolved in ACN/H_2_O (70:30, *v*/*v*) with 0.1% FA, and 1 mL was injected per run. The injection process was repeated eight times to obtain purified isoUA (Supplementary File, Figure S3). The purified isoUA was identified by comparing its retention time, UV spectrum and molecular mass with those obtained from the isoUA-producing reference lichen (i.e., *B. ramuliferum*) by metabolite profiling, as shown in Figure 3.

### 4.4. Metabolite Profiling

Our routine UHPLC-PDA-MS profiling started with lichen extracts, which were re-constituted from the acetone extract of ca. 15 mg lichen thallus materials [39,40]. Briefly, the intact lichen thalli were ground under liquid nitrogen and extracted twice with acetone. Then, the acetone was evaporated, and the dried residues were dissolved in methanol/ACN (50:50, *v*/*v*). An aliquot of the reconstituted solution was diluted 20 times with 50% ACN in H_2_O and filtered before HPLC-PDA-MS profiling. One Kinetex F5 column (3 × 150 mm, 2.6 µm; Phenomenex, Macclesfield, UK) was used for the separation of the UA and isoUA isomers. An isocratic elution was used under ambient temperature (22 ± 1 °C) at 0.45 mL/min flow rate, and the mobile phase consisted of H_2_O:ACN (25:75, *v*/*v*) with 0.1% FA. The UV spectrum was recorded from 200 to 500 nm, and the MS full scan was monitored from a 200 to 800 mass to charge ratio (*m*/*z*) in the negative ion mode. The isomeric UA and isoUA were monitored at the same molecular ion at 343 *m*/*z*, and their resolution primarily depended on chromatographic separation.

## 5. Conclusions

Overall, our study found that isoUA is produced in a very low quantity (24 ppm) in the well-characterized, DNA-barcoded *Cladonia arbuscula* from Iceland. This low quantity may therefore easily be overlooked during routine metabolite profiling. Its presence and identity were confirmed by UPLC-PDA-MS analysis after vigorous chromatographic purification steps. The characteristic UV spectrum obtained for isoUA could serve as a key spectroscopic identification marker in future studies. Our study emphasizes the need for more sensitive methods in lichen chemotaxonomy when chemical markers are present in extremely low quantities and highlights the value of integrating DNA barcoding with metabolite profiling in lichen chemistry research.

## Data Availability

Data will be made available on request.

## Supplementary Materials

The following supporting information can be downloaded at https://www.mdpi.com/article/10.3390/molecules31010143/s1, Figure S1: Neighbor-joining tree using the fungal nrITS alignment. Bootstrap values over 70% are labelled. The Icelandic specimen used for the isolation of isousnic acid is marked in bold, belonging to the *Cladonia arbuscula* s.l. clade recognized by Piercey-Normore et al.; Figure S2: PuriFlash chromatography for the purification of isousnic acid. The major peak is usnic acid eluting around 18 min, and the effluents eluting at the purple and pink zones were collected for isousnic acid; Figure S3: Prep-HPLC chromatograph showing the separation of usnic acid (19.17 min) and isousnic acid (21.41 min); Figure S4: Chromatograms of purified isousnic acid (upper) and the reference lichen *B. ramuliferum* (bottom), which contains isousnic acid as the major metabolite; Figure S5: High-resolution mass spectrum of purified isousnic acid. The deprotonated molecular ion is 343.0830 *m*/*z*.

## Abbreviations

The following abbreviations are used in this manuscript:

ACN	Acetonitrile
FA	Formic acid
HPLC	High-performance liquid chromatography
IsoUA	Isousnic acid
nrITS	Nuclear ribosomal internal transcribed spacer
RBP2	RNA polymerase II second largest subunit
s.l.	*Sensu lato* (in the broad sense)
s.str.	*Sensu stricto* (in the strict sense)
ssp.	Subspecies
UA	Usnic acid
UHPLC-PDA-MS	Ultra-high-performance liquid chromatography–photodiode array detection–mass spectrometry
