# Supplementary material for: Is the Reindeer Lichen Cladonia arbuscula Really Producing Isousnic Acid? A Chemotaxonomy Query
Source: Molecules. 2026 Jan 1;31(1):143. doi: 10.3390/molecules31010143 (PMC12787162; doi:10.3390/molecules31010143)
Supplement: Supplementary file 1 [file molecules-31-00143-s001.zip › molecules-4036993-supplementary.pdf]

Supplementary files

Is the reindeer lichen *Cladonia arbuscula* really producing isousnic acid? A chemotaxonomy query

Dagmar Ísleifsdóttir [1], Maonian Xu [1], Maia Biwersi [1], Marie-Jeanne Leblanc [2], Starri Heiðmarsson [3], Snæbjörn Pálsson [4], John L. Sorensen [2], Elvar Örn Viktorsson [1], Elín Soffía Ólafsdóttir [1,*]

[1] Faculty of Pharmaceutical Sciences, University of Iceland, Hofsvallagata 53, IS-107 Reykjavik, Iceland

[2] Department of Chemistry, University of Manitoba, Winnipeg, Manitoba, R3T 2N2, Canada

[3] Northwest Iceland Nature Research Centre, Aðalgata 2, IS-550 Sauðárkrókur, Iceland

[4] Faculty of Life and Environmental Sciences, University of Iceland, Sturlugata 7, 102 Reykjavik, Iceland

*Corresponding author:

Elín Soffía Ólafsdóttir, PhD, Professor

Email: elinsol@hi.is

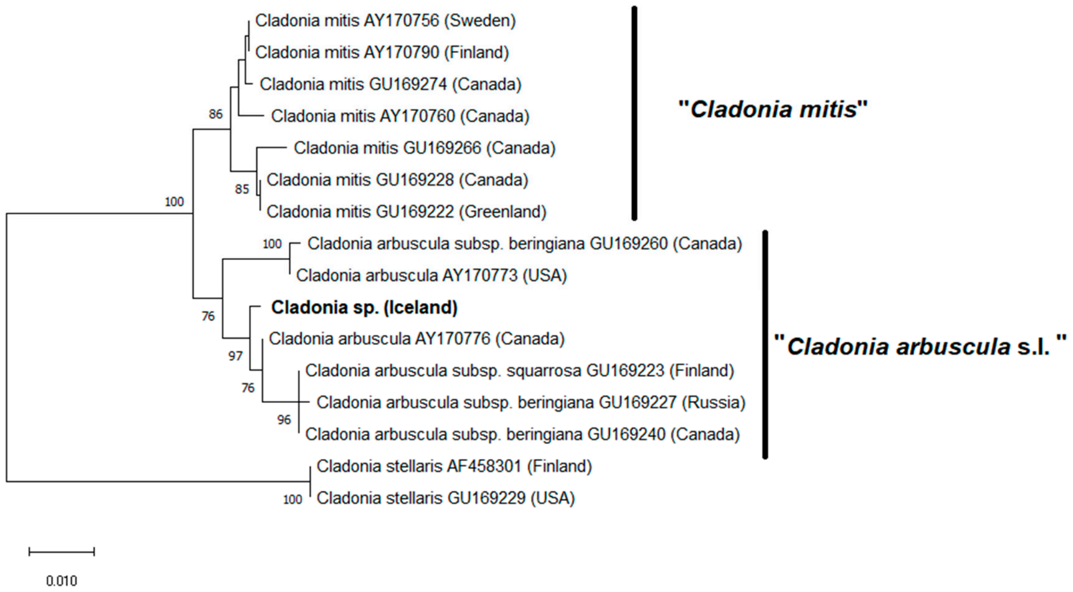

Figure S1. Neighbor-joining tree using the fungal nrITS alignment. Bootstrap values over 70% are labelled. The Icelandic specimen used for the isolation of isousnic acid is marked in bold, belonging to the *Cladonia arbuscula* s.l. clade recognized in Piercey-Normore *et al*. [1].

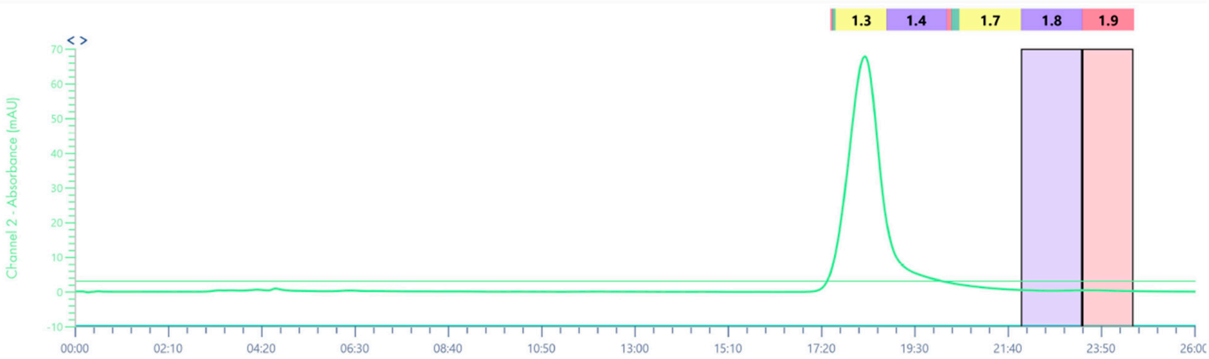

Figure S2. PuriFlash chromatography for purification of isousnic acid. The major peak is usnic acid eluting around 18 min, and the effluents eluting at the purple and pink zones were collected for isousnic acid.

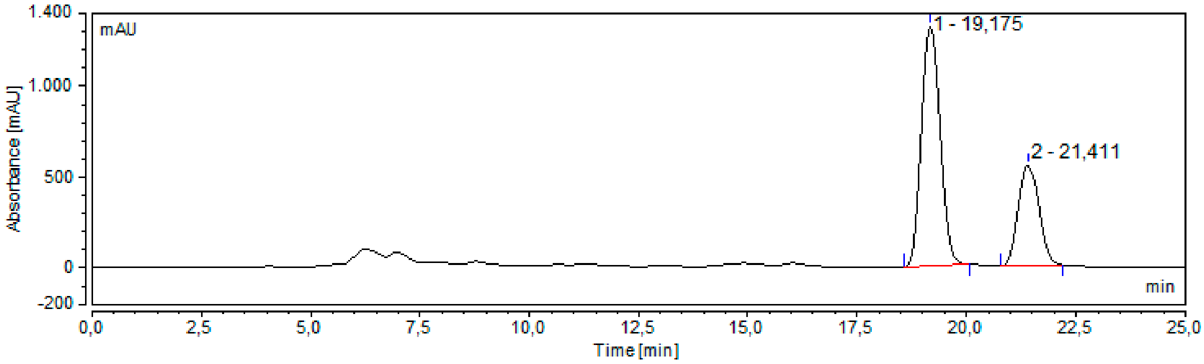

Figure S3. Prep-HPLC chromatograph show the separation of usnic acid (19.17 min) and isousnic acid (21.41 min).

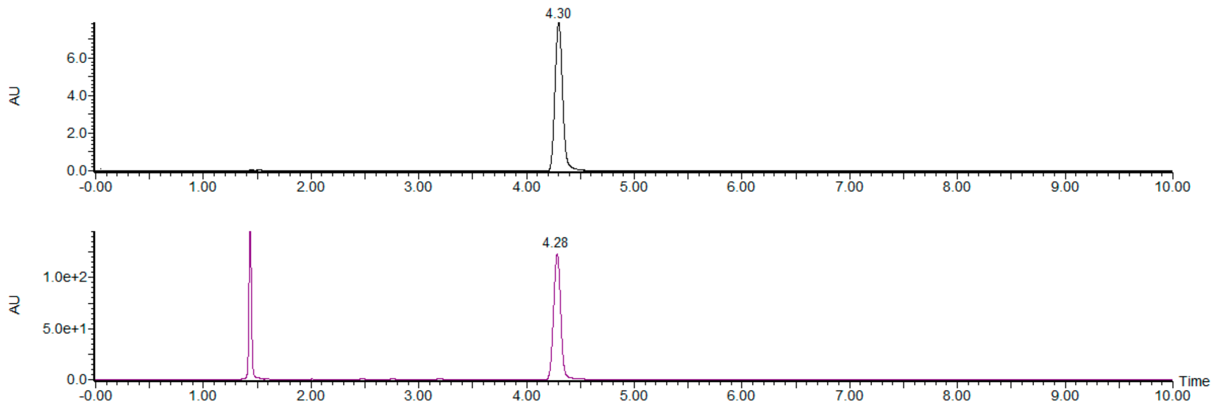

Figure S4. Chromatograms of purified isousnic acid (upper) and the reference lichen *Bunodophoron ramuliferum* (bottom) which contains isousnic acid as the major metabolite.

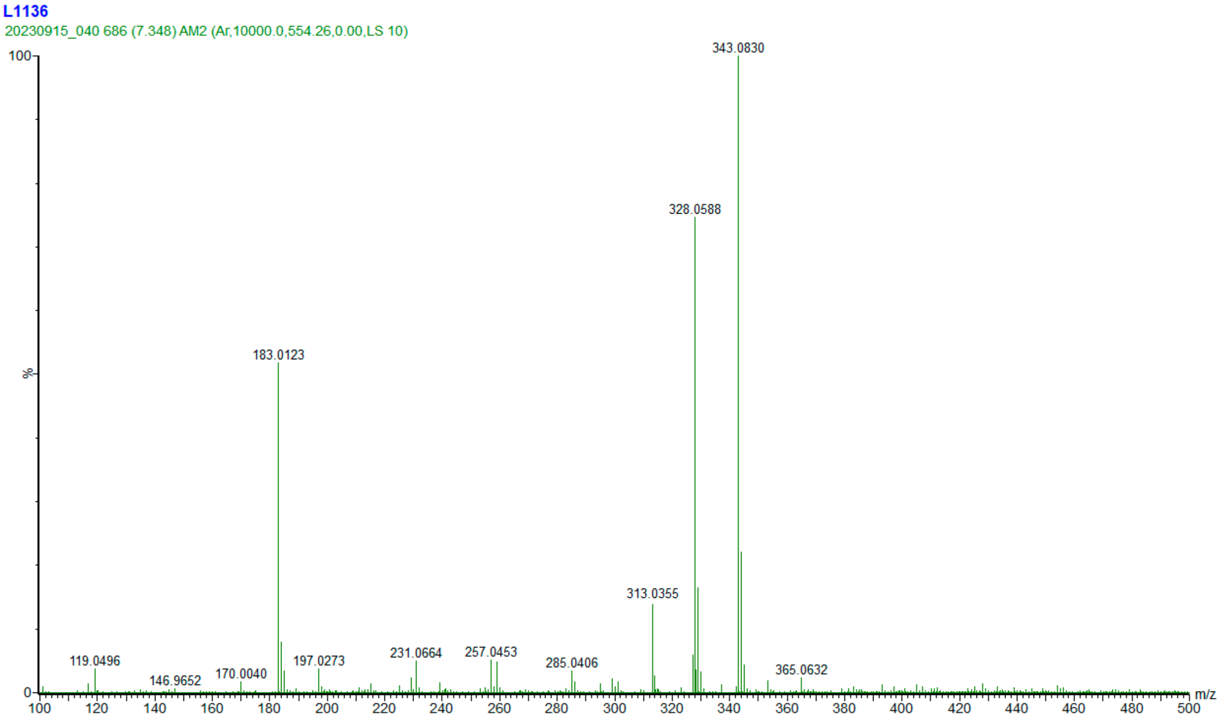

Figure S5. High-resolution mass spectrum of purified isousnic acid. The deprotonated molecular ion is 343.0830 m/z.

References

1. Piercey-Normore, M.D.; Ahti, T.; Goward, T. Phylogenetic and haplotype analyses of four segregates within Cladonia arbuscula s.l. *88*, 397-408, doi:10.1139/B10-027.
